# Scientists’ warning of the impacts of climate change on mountains

**DOI:** 10.7717/peerj.14253

**Published:** 2022-10-24

**Authors:** Jasper Knight

**Affiliations:** School of Geography, Archaeology & Environmental Studies, University of the Witwatersrand, Johannesburg, South Africa

**Keywords:** Climate change impacts, Anthropocene, Mountain environments, Deglacierization, Geohazards, Adaptation, Human impacts

## Abstract

Mountains are highly diverse in areal extent, geological and climatic context, ecosystems and human activity. As such, mountain environments worldwide are particularly sensitive to the effects of anthropogenic climate change (global warming) as a result of their unique heat balance properties and the presence of climatically-sensitive snow, ice, permafrost and ecosystems. Consequently, mountain systems—in particular cryospheric ones—are currently undergoing unprecedented changes in the Anthropocene. This study identifies and discusses four of the major properties of mountains upon which anthropogenic climate change can impact, and indeed is already doing so. These properties are: the changing mountain cryosphere of glaciers and permafrost; mountain hazards and risk; mountain ecosystems and their services; and mountain communities and infrastructure. It is notable that changes in these different mountain properties do not follow a predictable trajectory of evolution in response to anthropogenic climate change. This demonstrates that different elements of mountain systems exhibit different sensitivities to forcing. The interconnections between these different properties highlight that mountains should be considered as integrated biophysical systems, of which human activity is part. Interrelationships between these mountain properties are discussed through a model of mountain socio-biophysical systems, which provides a framework for examining climate impacts and vulnerabilities. Managing the risks associated with ongoing climate change in mountains requires an integrated approach to climate change impacts monitoring and management.

## Introduction

There is increasing concern about Earth’s biophysical systems and sustainability in the light of ongoing anthropogenic climate change (global warming). To this end, world scientists have sent a Warning to Humanity regarding the impacts of climate change on different physical systems and environments (*e.g*., Ripple et al., 2017; Finlayson et al., 2019; Albert et al., 2021). This article contributes to this debate by sending a Warning to Humanity on the impacts of climate change on mountain environments globally and the multifaceted, interlinked and long-lasting nature of these effects on both mountain physical environments and on people and communities. This Warning to Humanity confirms and extends the findings of the IPCC Special Report on the cryosphere that shows that, in mountains, there is high confidence that climate change has decreased snowcover, glacier mass balance and permafrost area (Hock et al., 2019b). In addition, IPCC Assessment Report 6 evaluates climate change impacts on mountains, and states with high confidence that climate change has “observable and serious consequences” for mountain ecosystems and communities (Adler et al., 2022).

Mountains represent an important physical environment, with 15.38% of the global land surface lying above 1,000 m asl, and 7.67% lying above 2,500 m asl (calculated from Owens & Slaymaker, 2004, their Table 1.3). The IPCC attributes the causes of present-day climate change in mountains to increasing greenhouse gas emissions, leading to anthropogenic global warming (Hock et al., 2019b; Adler et al., 2022). Field observations and measured data providing evidence for the effects of anthropogenic global warming in mountains, according to the IPCC, include: a decrease in snow cover at low elevations (high confidence), sustained negative glacier mass balance (very high confidence), a decrease in mountain permafrost area (high confidence), changes in the spatial patterns and timing of natural hazards (high confidence), changes in seasonality and volume of mountain river discharge (very high confidence), and changes in ecosystem composition (very high confidence). In detail, the close link of global warming (*i.e*., temperature change) to mountains comes about largely through the presence of snow and ice which has an important role in the regional heat balance through albedo feedbacks (Knight & Harrison, 2022). Here, light-toned snow and ice surfaces have high albedo, reflecting incoming solar radiation back out to space and keeping the land surface cool (Kokhanovsky et al., 2018). By contrast, dark-toned rock surfaces absorb radiation and therefore warm up, and this can trigger further snow and ice melt. Decreased snow cover and increased supraglacial debris on glaciers, however, can also dramatically increase the rate of mountain warming, especially where snowline elevation is rising (You et al., 2020). This climate amplification found in mountains, known as elevation-dependent warming, has been identified in many mountain blocks worldwide. For example, in the Tibetan Plateau, warming from the 1950s onwards across a range of stations averages 0.31 °C/decade^−1^ with values from the 1980s onwards between 0.50–0.67 °C/decade^−1^ (Kuang & Jiao, 2016). This compares with averaged global surface temperature increases from the 1980s onwards of 0.18 °C/decade^−1^ (NOAA, 2022), meaning temperatures are amplified by around a factor of three in mountains.

Anthropogenic climate change in mountains does not just affect temperatures. Changes in regional weather patterns are also observed, and these reflect the operation of synoptic atmospheric circulation patterns which are also changing under global warming (Letcher & Minder, 2018; Thornton et al., 2021). Associated with these patterns are variations in wind direction, humidity and development of an inversion layer caused by changes in the environmental lapse rate found in mountains—a result of changing ecosystems, soil moisture and snow/ice (Barry, 1992; Pepin et al., 2017; Hiebl & Schöner, 2018). In the European Alps, several studies have examined the climatology of rainfall patterns based on regional weather records from the 1960s onwards, and these show changes in both spatial precipitation patterns and long term precipitation trends that reflect the role of synoptic circulation interacting with topography (Frei & Schär, 1998; Isotta et al., 2014). Changing precipitation patterns mainly reflect windward—leeward effects (and therefore wind direction) rather than just variations by altitude. Studies have also explicitly linked variations in snow distribution, timing and depth over mountains to atmospheric circulation patterns, based on both observational data and climate models (Brown & Petkova, 2007; Letcher & Minder, 2018; Matiu et al., 2020). This shows the role of different atmospheric drivers in determining mountain precipitation patterns (*e.g*., position of blocking highs, strength of the North Atlantic Oscillation). Thornton et al. (2021, their Fig. 2) collate together all of the different mountain climate variables that are changing under anthropogenic climate change, that were identified according to an evaluation by experts undertaken through a Delphi process. Based on the classification of answers received from the expert panel (*n* = 837), 26.9% of answers correspond to the atmosphere alone (*i.e*., aerosols, greenhouse gases), 14.0% to the biosphere, 12.6% to the cryosphere, and 10.7% to the hydrosphere (classifications made by Thornton et al., 2021, calculations made from their Supplementary File S1). The modal class of answers (35.7%) corresponds to items such as precipitation, temperature and albedo that integrate all four ‘spheres’. This highlights that ongoing anthropogenic climate change is affecting many different elements of mountain climates.

Globally, mountain systems are currently undergoing rapid, significant and likely permanent change (Gerrard, 1991; Marston, 2008; Messerli, 2012; Hock et al., 2019b; Thornton et al., 2021). These changes are manifested in the physical properties of mountains and their dynamic behaviour, including mountain climate, geomorphology and ecosystems, and are described below. For example, decreases in mountain glacier volume and extent over the last decades are unprecedented in the wider context of the late Holocene (Zemp et al., 2015; Cogley, 2016; Beniston et al., 2018; Veettil & Kamp, 2019). Changes in mountain glaciers as a result of anthropogenic climate change have potential to impact on the workings of mountain physical systems as a whole (Adler et al., 2022) and to give rise to severe negative impacts on people and the environment through hazards and changes in environmental resources and services (Muccione, Salzmann & Huggel, 2016; Klein et al., 2019). In addition, the effects of climate change in mountains can also be amplified by different human activities taking place in these sensitive environments, such as agriculture, urbanization, land use change, mining and tourism (Hossain et al., 2020; Payne et al., 2020). This highlights that appropriate management and adaptation strategies to reduce risk and impacts are critical to sustainable human activity in mountains.

Mountains also represent important scenic and heritage landscapes because of the common presence of rare ecosystems, endemic species, and indigenous communities and cultural practices (Debarbieux & Price, 2008, 2012; Rasul & Molden, 2019; Chakraborty, 2021; Thornton et al., 2021). The close genetic relationship between these properties means that mountains can be considered as integrated biosystems, describing the interplay of climate, physical processes, ecosystems and people (*e.g*., Nowak, Nowak & Nobis, 2014; Stanisci et al., 2016; Allegrezza et al., 2017). Globally, these biosystems are now operating beyond their natural planetary boundaries because of their sensitivity to radiative forcing and their land surface feedbacks in response to forcing (Nogués-Bravo et al., 2007; Pepin & Lundquist, 2008; Huggel et al., 2010). Direct human interventions in mountains such as by agriculture and infrastructure development can also lead to these systems experiencing feedbacks, such as where land use change and deforestation results in enhanced soil erosion (Arnaud et al., 2016; Berteni & Grossi, 2020). Recognising this, the United Nations’ “International Year of the Mountains” was declared in 2002 (Ives & Messerli, 1999), and the “International Year of Sustainable Mountain Development” was declared in 2022 (Romeo, Manuelli & Abear, 2022).

The concept of sensitivity is also important when considering the present and future responses of mountain systems to climate change and other anthropogenic forcings. Climate sensitivity is a concept used in climate models and refers to the atmospheric temperature response to changing levels of atmospheric CO_2_ (Shindell, 2014). A variant of this concept, termed equilibrium climate sensitivity (ECS), refers to the temperature response that arises as an outcome from the operation of Earth’s geomorphological, hydrological and biological systems, following forcing by CO_2_ (Knutti, Rugenstein & Hegerl, 2017). ECS is therefore a more accurate reflection of the integrated Earth system response to anthropogenic climate forcing (Knight & Harrison, 2013), and this concept can be applied to understand how the mountain cryosphere, hydrosphere and biosphere (as defined in mountains by Thornton et al., 2021) respond to anthropogenic climate forcing. Broadly, higher sensitivity means that a system responds more quickly and dynamically to forcing; lower sensitivity means a system responds more slowly or with a more subdued expression (Previdi et al., 2013). Several studies have examined the sensitivity of the mountain *cryosphere* (snow, glacier ice, permafrost) (Knight & Harrison, 2022). The sensitivity of snow is measured according to its heat balance effects (albedo) using the units W m^−2^ K^−1^. The sensitivity of glaciers is measured in terms of mass balance change using the units m w.e. (water equivalent) yr^−1^ K^−1^. Lowland permafrost sensitivity is usually measured through km^2^ area change per K^−1^ but the same approach is less meaningful for mountain permafrost because of the varying relief, altitude and hypsometry of different mountains (Slater & Lawrence, 2013). Thus, the concept of sensitivity of the mountain cryosphere is multifaceted with the major control being temperature but precipitation and the properties of the land surface also being important. Sensitivity of the mountain *hydrosphere* is usually described in terms of changes in river runoff in response to climate change (including temperature, precipitation, and snow/ice melt) (Shi & Durran, 2014; Zhang et al., 2020). Different measures of this ‘sensitivity’ have therefore been used, including peak, seasonal or annual discharge variations, varying snow/glacier melt contributions, timing of peak flow, groundwater recharge *etc*. (*e.g*., Barnhart, Tague & Molotch, 2020; Zhang et al., 2020). This means that calculations of hydrosphere ‘sensitivity’ are location-specific and may not be comparable to other mountain river systems. Changes in water availability on steep mountain slopes, along with ongoing glacier retreat and paraglacial relaxation, has implications for mass movements, soil erosion and fluvial sediment yield, termed geomorphological sensitivity (Knight & Harrison, 2013, 2014, 2018; Rathburn, Shahverdian & Ryan, 2018). This is associated with land surface (geomorphological) change and geological hazards. Geomorphological sensitivity in mountains has commonly been evaluated through reconstructing the timing and magnitude of past hazard events using dating, sedimentary and geomorphological evidence (Keiler, Knight & Harrison, 2010; Fischer et al., 2012; Kirschbaum, Stanley & Zhou, 2015), but this evidence may not be present in all mountains, and not every mountain block has been studied in this way. This means there is incomplete understanding of mountain geomorphological sensitivity. Several studies have examined the responses of plant ecosystems or individual species to climate change in mountains, mainly in terms of bioclimatic niches and extinction risk based on future climate scenarios (*e.g*., Chakraborty, Joshi & Sachdeva, 2016; Dagnino et al., 2020; Xu et al., 2020). The results of such studies of *biosphere* sensitivity focus on changes in net primary productivity and phenological patterns across mountains and identifying potential changes in areal extent and species range for the specific mountains examined. Quantitative modelling approaches using different spatial and temporal ecological datasets across large regions have also been developed (Gao, Jiao & Wu, 2018; Kling et al., 2020) but these have not been widely applied to mountains, especially at a smaller scale. The role of direct human activities on mountain ecosystems through agriculture, urbanization and invasive species has not been considered in these models.

This overview of mountain system sensitivity highlights several key points: (1) The different physical elements that are present within mountains (snow/ice, mountain slopes/soil, vegetation) exhibit different sensitivities to climate as well as likely to other environmental and anthropogenic forcings, although this is not fully understood; (2) ‘sensitivity’ of these different elements is interpreted and quantified in different ways, meaning that deriving an overview of the sensitivity of any mountain system in totality is problematic; (3) it is not always clear how these mountain elements are going to evolve under future climate change, given their varying sensitivities to forcing; and (4) human activities taking place in mountains is already changing—and will continue to change—different mountain elements, which means that their calculated sensitivities to climate forcing may bear little relation to their actual future changes, if human activity is a more dominant control on their dynamics. Mountains are thus complex integrated systems and may respond to future climate change in ways that are not fully understood or which have low predictability. This has implications for identifying and managing future risks associated with hazards, water supply, and ecosystem and cultural services.

Various lines of evidence, described below, from mountain blocks worldwide reveal the impacts of anthropogenic climate change on mountain processes, properties and communities. This study presents a Warning to Humanity on the negative and likely irreversible impacts of anthropogenic climate change on mountain environments worldwide. This is informed by evidence of contemporary and past changes in mountain systems, and by climate model outputs reported in the literature that predict future changes in precipitation, temperature, snow and permafrost properties, and glacier mass balance. These then in turn have implications for mountain biophysical processes, ecosystems, resource types and availability, and human activity. A significant result of the analysis in this study is that mountain systems are confirmed to be highly vulnerable, and thus exhibit high sensitivity, to anthropogenic climate change and that, from almost all perspectives, negative outcomes to the physical and human environments are anticipated, and are indeed already taking place.

This study identifies and discusses the impacts of climate change on four key properties of mountain systems (including aspects of human activity), which provides an interpretive framework for a better understanding of mountain system evolution in the Anthropocene. The specific terms used in this study focusing on hazards, risk and resilience follow IPCC Assessment Report 5 definitions (IPCC, 2014).

## Survey methodology

Much work on mountains globally is site-specific and often deals with only certain aspects of the biophysical environment, in particular the changing cryosphere. There are fewer studies that have focused on mountain communities and their use of environmental and climate-related resources. However, relationships between different mountain system elements have not been examined in detail, from either individual mountain blocks or from across different climatic or geologic settings. This is a limitation in identifying globally-applicable relationships between mountain system elements, and thus in building biophysical system models to explain the impacts of climate forcing. The aim of this study is to integrate evidence from examples globally on mountain system properties and dynamics, and derive an overarching analysis of mountains as biophysical systems. To achieve this, relevant peer-reviewed published literature was identified from ISI Web of Science using the search term of “mountain systems” and then the results refined based upon the search term “climate change”. The resulting literature was included where it considered relationships between different mountain properties as developed in specific case studies. Therefore, the literature examined focuses on quantitative studies that examine the cause-and-effect relationships between mountain properties. The co-relationships between different mountain properties, and their dynamics, are then used in this study as the basis for developing a new socio-biophysical model for mountain systems. This provides a powerful way of conceptualizing both the integrated workings of mountain systems, and the potential sensitivity of these systems to climate forcing in the Anthropocene, and thus why this sends a Warning to Humanity of climate change impacts on mountain environments.

## Results

**Figure 1:**
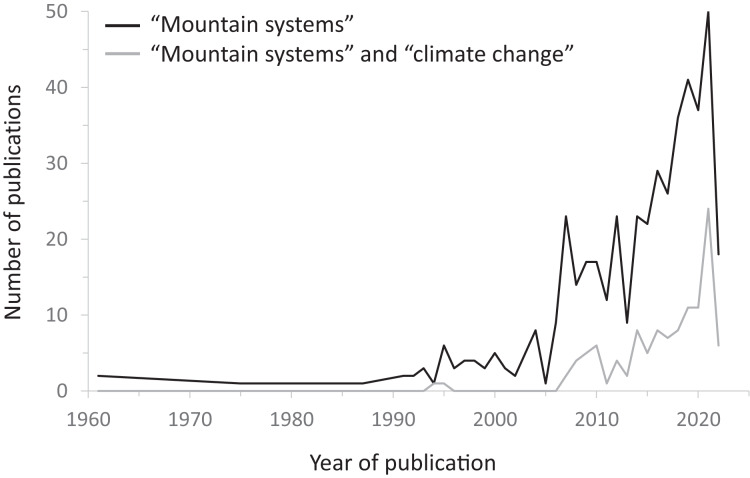
Graph showing the number of published articles from the Web of Science database (accessed 30 July 2022) according to year of publication, using different search terms.

From the Web of Science literature search, 464 individual articles were identified using the search term “mountain systems” (Table 1), and 39% of all these papers were published in the last 5 years (2018–2022). The earliest publications including such a term date from 1961. A similar temporal pattern is seen with the search terms “mountain systems” and “climate change” where 44% of all papers come from the last 5 years. It is notable that in all instances there is a big increase in the number of studies on mountain systems in the last 15 years (2008–2022; Fig. 1). These publications were also examined for their Web of Science category of academic discipline (Table 2). Although this classification is only indicative, it shows that the most common academic fields of “mountain systems” are in ecosystems (Ecology/Plant Sciences/Zoology/Biodiversity Conservation; cumulatively 31% if the total), the physical landscape (Geosciences Multidisciplinary/Geography Physical; cumulatively 15% of the total), and Environmental Sciences (11%). Including the search term “climate change”, a slightly different pattern emerges with, in percentage terms, a greater emphasis on Ecology, Environmental Sciences, Biodiversity Conservation, Meteorology Atmospheric Sciences, and Environmental Studies (Table 2). This shows the greatest areas of research interest in climate change in mountains, focusing on climate patterns/predictions and ecosystem responses. Only in Plant Sciences is there significant under-representation with “climate change” (3.7%) compared to without it (6.7%). Based upon the literature search results, four major mountain properties are identified according to the dominant focuses of individual research studies (glaciers and permafrost related to the mountain cryosphere; mountain hazards and risk; mountain ecosystems; mountain communities and infrastructure). These properties and their dynamics are now discussed.

**Table 1:** Literature search results from the Web of Science (accessed 30 July 2022) using different search terms, according to year of publication (see Fig. 1). The earliest items appearing in the search results were published in 1961.

Year of publication	Web of Science category for the search term “mountain systems”	Web of Science category for the search term “mountain systems” and “climate change”
2022	18	6
2021	50	24
2020	37	11
2019	41	11
2018	36	8
2017	26	7
2016	29	8
2015	22	5
2014	23	8
2013	9	2
2012	23	4
2011	12	1
2010	17	6
2009	17	5
2008	14	4
2007	23	2
2006	9	0
2005	1	0
2004	8	0
2003	5	0
2002	2	0
2001	3	0
2000	5	0
1999	3	0
1998	4	0
1997	4	0
1996	3	0
1995	6	1
1994	1	1
1993	3	0
1992	2	0
1991	2	0
1990	0	0
1989	0	0
1988	0	0
1987	1	0
1986	0	0
1985	0	0
1984	0	0
1983	0	0
1982	0	0
1981	1	0
1979	1	0
1978	0	0
1977	0	0
1976	0	0
1975	1	0
1961	2	0
**Total**	**464**	**114**

**Table 2:** Categorisation of search results from the Web of Science database (accessed 30 July 2022). Note that individual published articles in the database may be classified under several categories. Categories with fewer than five and three published articles for “mountain systems”, and “mountain systems” and “climate change”, respectively, are not included in the table. Items in italics indicate where there is a significantly higher value recorded between the two columns of results.

Web of Science categories	Web of Science category for the search term “mountain systems” (% of total)	Web of Science category for the search term “mountain systems” and “climate change” (% of total)
Ecology	102 (13.7%)	*36 (16.8%)*
Environmental sciences	79 (10.6%)	*39 (18.2%)*
Geosciences multidisciplinary	58 (7.8%)	16 (7.5%)
Geography physical	54 (7.2%)	15 (7.0%)
Plant sciences	*50 (6.7%)*	8 (3.7%)
Evolutionary biology	46 (6.2%)	13 (6.0%)
Zoology	*44 (5.9%)*	6 (2.8%)
Biodiversity conservation	35 (4.7%)	*16 (7.5%)*
Multidisciplinary sciences	28 (3.7%)	6 (2.8%)
Meteorology atmospheric sciences	27 (3.6%)	*11 (5.1%)*
Biochemistry molecular biology	16 (2.1%)	3 (1.4%)
Geography	16 (2.1%)	5 (2.3%)
Entomology	14 (1.9%)	
Forestry	14 (1.9%)	4 (1.8%)
Genetics heredity	14 (1.9%)	
Soil science	13 (1.7%)	
Water resources	13 (1.7%)	6 (2.8%)
Environmental studies	10 (1.3%)	*8 (3.7%)*
Geochemistry geophysics	10 (1.3%)	
Biology	9	3 (1.4%)
Ornithology	7	
Geology	6	
Green sustainable science technology	6	3 (1.4%)
Marine freshwater biology	6	
Oceanography	6	
Imaging science photographic technology	5	3 (1.4%)
Remote sensing	5	
(Other categories)	52 (7.0%)	13 (6.0%)
**Total**	**744**	**214**

### The mountain cryosphere

#### Mountain glaciers

As a consequence of global warming, mountains glaciers worldwide including ice caps, valley and cirque glaciers are undergoing a trajectory of enhanced melt and thus negative mass balance over recent decades (*e.g*., Cogley, 2016; Azam et al., 2018; Cao et al., 2019; Ding et al., 2020). The result of this can be seen through (1) long-term changes in glacier area or spatial extent; (2) changes in glacier volume as expressed through mass balance; and/or (3) changes in glacier dynamics, as evidenced by oscillations of the glacier margin. As such, glacier responses to climate forcing can be diverse, and expressed differently according to topographic setting, elevation, climate, and glacier size. Mountain glaciers are generally sensitive to temperature changes due to their relatively small size and steep surface gradient (Bach, Radić & Schoof, 2018; Bolibar et al., 2022). This is because subtle variations in temperature, driving glacier mass balance, can result in changes in the position of the equilibrium line altitude (ELA) which, globally, is rising due to climate change (Six & Vincent, 2014; Lorrey et al., 2022). Vargo et al. (2020) used glacier mass balance modelling of glaciers in the Southern Alps (New Zealand), based on temperature and precipitation outputs from climate models. They showed that anthropogenic climate forcing increases the likelihood of extreme glacier mass loss by six to 10 times. Several studies have also projected glacier ELA and thus mass balance responses across mountain blocks (*e.g*., Liu et al., 2019; Žebre et al., 2021; Lorrey et al., 2022) but in detail these responses are highly spatially variable. This may reflect both differing sensitivity of climate by ice masses of different sizes (Bach, Radić & Schoof, 2018), but also microclimate effects which are particularly significant in areas of high local relief such as mountains (Rankl, Kienholz & Braun, 2014; Six & Vincent, 2014). This is highlighted by cryospheric models that suggest an over-reliance on temperature as a forcing factor in mountain glacier response (Bolibar et al., 2022), rather than consider system feedbacks such supraglacial debris cover, snow depth, and wind-transported snow as factors influencing glacier mass balance (Dobhal, Mehta & Srivastava, 2013). Although mountain glaciers have responded to climate changes throughout the Holocene, monitoring using field and remote sensing data over recent decades shows the imprint of global warming on the state of the mountain cryosphere (*e.g*., Banerjee & Shankar, 2013; Huss et al., 2017; Beniston et al., 2018; Hock et al., 2019b; Gärtner-Roer et al., 2019). Such studies also highlight the spatial and temporal variability of mountain glacier responses depending on their altitude, aspect, size and ELA (Dehecq et al., 2019). This is also reflected in future modelled projections of glacier volume and area change that show, for example, that different sectors of Tibetan Plateau mountains have projected volume loss rates of −0.06 to −1.90% yr^−1^, and area loss rates of −0.21 to −1.85% yr^−1^ between 2000 and 2050 (Zhao, Ding & Moore, 2014).

Many regional studies of historical mountain glacier changes, using a combination of field and remote sensing data, have been undertaken. These studies can inform on the rate and style of glacier change and link these derived parameters to climate forcing or coeval changes in environmental regimes in the local area. For example, Landsat and Sentinel-2 data in the Bolivian Andes show glacier area reduction of 51% between 1975 and 2016 (1.20% yr^−1^), with the least change recorded for glaciers located above 5,500 m asl (Veettil et al., 2018). This compares with a decrease in glacier area by an average of −0.57% yr^−1^ (1960–2010) over High Mountain Asia, but with high spatial variability with some 65% of datapoints statistically identical to zero change (Cogley, 2016). In the western Himalayas region (1977–2016) Landsat data show that the snow line elevation increased by 116 ± 17 m, glaciers decreased in area (by 6.25 ± 0.0012% or 0.16% yr^−1^), average glacier snout recession rate increased (from 16 ± 3.4 m yr^−1^ in 1977 to 23 ± 3.4 m yr^−1^ in 2016), and glacier debris cover area increased by 80% (Shukla et al., 2020). In the Karakoram, Landsat data (1976–2012) show that 79% of glacier termini were stable, 5% advanced, 8% retreated, and 8% belong to oscillating (surging) glaciers (Rankl, Kienholz & Braun, 2014), confirmed by more recent mass balance studies (Farinotti et al., 2020). Glaciers across China show a long-term average mass balance decrease of −0.0135 m w.e. yr^−1^ (1960–2019) with the longest (1959–2019) record from Urumqi Glacier No. 1 showing a decrease of −0.0142 m w.e. yr^−1^ (Su et al., 2022). All these values were statistically significant (*p* < 0.0001). By contrast, for High Mountain Asia as a whole based on ASTER DEMs, average glacier mass balance change in the period 2000–2016 was −0.18 ± 0.04 m w.e. yr^−1^ (range +0.14 to −0.62 m w.e. yr^−1^) (Brun et al., 2017). These studies provide a snapshot of individual glaciers, over different time periods and using different methodologies but implications of the trajectories of glacier change for the wider mountain environments of these localities are not commonly discussed.

These studies and others highlight that responses of individual glaciers to climate change in different mountain massifs are highly variable, likely due to microclimate effects and feedbacks (Huss & Fischer, 2016; Azam et al., 2018; Baldasso et al., 2019; Carturan, Rastner & Paul, 2020). Mutz & Aschauer (2022) show that the mass balance of different Andean glaciers is statistically related to different climatic variables including temperature, precipitation (both seasonal and annual), El Niño–Southern Oscillation and the Antarctic Oscillation, depending on glacier location. In addition, changing debris cover (thickness, debris size, distribution) is a critical influence on albedo and insulation effects, which can lead to marked reductions in glacier mass loss and frontal dynamics (Banerjee & Shankar, 2013; Dobhal, Mehta & Srivastava, 2013). These factors highlight that glacier mass balance does not solely reflect climate forcing because of the role of antecedent and geological factors. The multidecadal response times of many mountain glaciers also mean that they are likely out of mass balance equilibrium with prevailing climate, irrespective of their sensitivity to climate forcing (Christian, Koutnik & Roe, 2018). However, other studies have described a more deterministic relationship of mountain glaciers to temperature (Bolibar et al., 2022), with Geyman et al. (2022) showing—based on historical photogrammetry—a mass balance response of −0.28 m yr^−1^ per 1 °C temperature rise of Svalbard glaciers. Responses of mountain systems to deglaciation under climate change fall within the frame of paraglacial process regimes, and the nature of these responses in terms of slope and fluvial sediment yields have been examined from both late Quaternary and Anthropocene examples (*e.g*., Cossart & Fort, 2008; Scapozza, 2016). Such studies highlight that mountain systems undergo very rapid changes associated with ice retreat, and that these impacts are wide ranging with respect to ecosystems, geohazards, and mountain water and sediment yield (Knight & Harrison, 2014). Land surface models also show the changing sensitivities of glaciers, permafrost and mountain landforms to forcing through the paraglacial period, and this can help explain why mountain system responses to climate change may vary over time and space (Knight & Harrison, 2018). Field data, however, are not always interpreted in the context of such theoretical insights.

Climate models and historical trajectories of glacier mass loss have also been used to consider where, how and when mountain glaciers are likely to become functionally inactive, or melt completely, and the rate of water equivalent loss, under different climate change scenarios. For example, Hock et al. (2019a) used the four standard IPCC representative concentration pathways (RCPs) in order to consider regional glacier responses to future temperature patterns from 25 different GCMs. The predicted mass loss from different regions varies significantly according to glacier extent and type (lowland ice sheet *vs* mountain ice cap or cirque/valley), but all RCP scenarios show similar patterns until the mid-21^st^ century after which these patterns diverge. The models also predict a high glacier mass loss (commonly ~60–>90%) for many mountain blocks worldwide by 2100 under the RCP8.5 emissions scenario. A similar approach with similar results was also used by Shi et al. (2020) for the Tibetan Plateau.

Based on a global temperature rise of 1.5 °C by 2100 using Coupled Model Intercomparison Project Phase 5 (CMIP5) outputs and RCP2.6, high Asian mountains are predicted to warm by 2.1 ± 0.1 °C and result in a 36 ± 7% total mass loss (Kraaijenbrink et al., 2017). Values for other RCP scenarios are much higher, but with temperature and mass loss responses varying across different mountain sectors (*ibid*). More detailed regional studies also show complex glacier responses, such as in the European Alps where mountain glacier slope, topographic setting and debris cover control sensitivity to climate forcing (Huss & Fischer, 2016; Žebre et al., 2021). Such field data are confirmed across wider regions through monitored reference glaciers of the World Glacier Monitoring Service (https://wgms.ch/). These data show continuous mass balance loss in all global regions and at a rate that has increased over time (since 1950), to a volume of 0.98 m w.e. yr^−1^ and 0.77 m w.e. yr^−1^ in 2019/20 and 2020/21, respectively. Glaciological and climate models have also been used to predict the fate of individual glaciers. For example, modelling of Austre Lovénbreen, Svalbard, suggests rapid area and mass balance decrease, and highest meltwater yield, in the middle of the 21^st^ century, with the glacier wholly gone by 2120 (Wang, Lin & Ai, 2019). There are similar results using different RCP scenarios for Great Aletsch Glacier, Switzerland (Jouvet & Huss, 2019). However, such projections often use different model scenarios, different temporal starting points, and different input parameters and trajectories of temperature and precipitation. This means that such results may not be easily comparable. In addition, if there are glaciers of different sensitivities, then there may be a range of future glaciological responses (Carturan, Rastner & Paul, 2020; Bolibar et al., 2022) but these factors are not fully considered with respect to impacts on wider mountain systems.

#### Mountain permafrost

Mountains worldwide already show increased permafrost temperatures, both in the near-surface and at depth (Harris et al., 2003; Liu et al., 2017; Severskiy, 2017). Sensitivity analysis of arctic permafrost to warming suggests areal changes of 4.0 + 1.0/−1.1 million km^2^ per 1 °C of warming (Chadburn et al., 2017). The sensitivity of mountain permafrost to climate forcing is more difficult to establish because of mountains’ steep and topographically complex environments and microclimates. However, sensitivity analysis from finite element modelling highlights the roles of snow depth and mean annual air temperature (Luetschg, Lehning & Haeberli, 2008) and subsurface ice content and temperature (Noetzli et al., 2007; Scherler et al., 2013) on mountain permafrost stability.

Different field, remote sensing and modelling studies show the varied distributions and properties of permafrost in areas such as the European Alps (*e.g*., Boeckli et al., 2012; Deluigi, Labiel & Kanevski, 2017; Kenner et al., 2019) and the Tibetan Plateau/Himalayas (Gruber et al., 2017; Liu et al., 2017; Gao et al., 2021). Variations in active layer thickness and subsurface temperatures are key indicators of permafrost degradation used in monitoring studies (*e.g*., Hanson & Hoelzle, 2004; Pogliotti et al., 2015; Kellerer-Pirklbauer, 2019). Several studies also show that permafrost distributions and properties are influenced by local-scale and site-specific slope properties including subsurface moisture content, debris size, slope aspect, length and backwall height (*e.g*., Noetzli et al., 2007; Kellerer-Pirklbauer, 2019). There are also differences between active and relict permafrost, identified according to whether the slope is or is not undergoing creep, largely related to moisture availability rather than temperature. Therefore, the factors contributing to permafrost instability under climate change is more complex than just temperature forcing alone (Pogliotti et al., 2015; Gruber et al., 2017), and permafrost system sensitivity must therefore be set in a topographic and geomorphic context (Verleysdonk, Krautblatter & Dikau, 2011). In addition, information on permafrost thickness, distribution and temperature regime is unknown or is poorly reported in many mountain blocks worldwide, including in Africa, South America and the Middle East. This is a limitation on projections of future permafrost change and their impacts on some mountains, including the loss of geoheritage. Particular attention has also been paid to the monitoring of permafrost within rock bodies, in particular steep rock walls where permafrost degradation can result in rock slope failure (Gruber & Haeberli, 2007; Bodin et al., 2017; Keuschnig et al., 2017). This also includes the development of rock glaciers, formed as a result of interstitial permafrost or glacier ice present within a coarse clastic matrix (Knight, Harrison & Jones, 2019). Rock glaciers represent a distinctive signature of cryosphere decay in mountains, and these landforms are projected to increase in number and significance upon deglacierization in the Anthropocene (Knight & Harrison, 2014; Knight, Harrison & Jones, 2019).

The outcomes of climate warming on mountain permafrost include a rise in the lowest elevations at which permafrost is found; permafrost thinning and disaggregation; warming subsurface temperatures and thickening active layer; decreasing slope stability and increasing mass movement hazards (Gude & Barsch, 2005; Fukai et al., 2007; Bonnaventure & Lamoureux, 2013). The precise nature of permafrost responses depends on its depth, distribution and temperature. Under different RCP scenarios using the CMIP5 climate model, active layer thickness across northern hemisphere cold regions to 2100 is projected to increase between 0.77 ± 0.08 cm decade^−1^ (RCP2.6) and 6.51 ± 0.07 cm decade^−1^ (RCP8.5) (Peng et al., 2018). Irrespective of future warming rates, these projections are all significantly higher than reconstructed historical rates of 0.57 ± 0.04 cm decade^−1^ for the period 1850–2005 (*ibid*). In the Tibetan Plateau, CMIP5 modelling suggests permafrost area will decrease by 10.5% and 32.7% by 2040 and 2070, respectively, under the RCP8.5 scenario (Chang et al., 2018). Permafrost in the northwest Tibetan Plateau is likely to be most resilient to climate warming. More recent CMIP6 modelling using the updated IPCC shared socioeconomic pathway (SSP) 5–8.5 (equivalent to RCP8.5) suggests permafrost temperature in the Tibetan Plateau will increase by 2.6 ± 0.3 °C and active layer thickness by 3.0 ± 1.0 m by 2100 (Zhang et al., 2022). Based on a downscaled regional climate model (RCM), frost frequency in the Mont Blanc massif (French Alps) to 2100 is predicted to significantly decrease by 30–50%, depending on altitude, with implications for the rate and efficacy of physical weathering, permafrost melt, and land surface stability (Pohl et al., 2019). Similar future climate impacts on permafrost on other mountain massifs elsewhere in the world are not well understood.

### Mountain geohazards and risk

Mountains generally are areas of high hazard risk because of their common co-location with earthquakes and volcanoes, their steep slopes, harsh climate, and presence of snow and ice (Korup & Clague, 2009; He et al., 2012). This creates a challenging biophysical environment for human activity. Apart from geophysical hazards that are unrelated to climate, the melting of glaciers, permafrost and snow gives rise to land surface instability and mass movement hazards (Keiler, Knight & Harrison, 2010; Ding et al., 2020; Kirschbaum et al., 2020). Several studies have shown how these cryospheric hazards, individually and in combination, have been amplified in number and magnitude as a result of global warming (*e.g*., Stoffel, Tiranti & Huggel, 2014; Harrison et al., 2018; Ding et al., 2020; Stuart-Smith et al., 2021). However, there is significant spatial and temporal variability in such patterns (*e.g*., Schlögl et al., 2021; Heiser et al., 2022). A negative glacier mass balance, resulting in increased meltwater yield, can give rise to a range of land surface instabilities and geohazards. For example, runoff and sediment fluxes in the Tuotuohe River (part of the Yangtze River, Tibetan Plateau) increased by 135% and 78% from 1985–1997 to 1998–2016, respectively, as a result of enhanced cryosphere melt and increased precipitation (Li et al., 2020). Ouflowing rivers from deglacierizing catchments show an increase in discharge as a result of this higher water availability (Juen, Kaser & Georges, 2007; Tahir et al., 2011; Li et al., 2020). Further, this leads to changes in seasonality of maximum annual floods, with spring discharge corresponding to snowmelt freshets, and summer discharge corresponding to maximum glacier melt. Observation and modelling studies have been used to identify and then decouple different mountain water sources contributing to outflowing river discharge, and changes in total discharge over time and space and the balance between different water sources (Chen et al., 2017; Sanmiguel-Vallelado et al., 2017). This is because water availability may correspond to both melting glaciers and changes in precipitation regimes. Catchment and hydrological modelling studies show that cryosphere changes in addition to climate-driven changes in rainfall seasonality affect discharge patterns of mountain rivers, contributing to hazard risk (Huss et al., 2010; Mallucci, Majone & Bellin, 2019). Detection and attribution studies can inform on how these controls may change over time and space (Mallucci, Majone & Bellin, 2019). Glacial melting can also lead to the development of proglacial lakes and glacial lake outburst floods (GLOFs) (Harrison et al., 2018; Khadka, Zhang & Thakuri, 2018; Stuart-Smith et al., 2021). In Nepal, proglacial lakes have increased in number (by 181%) and area (by 82%) between 1997 and 2017 as a consequence of climate change, but these lakes vary significantly in their evolutionary trajectories depending on their elevation, topography, glacier size and local climate (Khadka, Zhang & Thakuri, 2018). GLOF size and recurrence interval likely show a lagged relationship to climate forcing (Harrison et al., 2018), although this has not been fully explored. GLOFs have been noted from several mountain blocks worldwide, and their potential for geohazard risk has been examined (Ahmed et al., 2021; Veettil & Kamp, 2021).

Glacier retreat and permafrost melting in combination lead to unstable land surfaces and enhanced mass movement activity. This genetic relationship has been noted from several mountain massifs (Sattler et al., 2011; Fischer et al., 2012; Haeberli, Schaub & Huggel, 2017) where several mass movement types can result, including landslides, rock slope failures, debris flows, colluvial fans and terraces, scree and talus, and rockfall. First, glacier melt leads to increased number and/or magnitude of flood events within mountain catchments, and this pattern has been noted with respect to climate forcing over different timescales and affecting glacier and snowpack melt regimes (Yao et al., 2007; Schulte et al., 2015). In the Himalayas, river hydrology varies spatially according to the contribution of monsoon rainfall, snow or glacier melt to river discharge, and this meltwater contribution also varies throughout the year (Qazi et al., 2020). Increased water availability on and beneath the land surface can then lead to rockfalls, landslides, debris/mudflows (He et al., 2012; Stoffel, Tiranti & Huggel, 2014; Kirschbaum et al., 2020), or avalanches within thicker or warmed snowpacks (Muntán et al., 2009). Analysis of dated mass movements of different types through the period of the European Little Ice Age (LIA, ~1550–1850 AD) shows that landslides are more common earlier in the LIA (~1660 AD), with the peak of avalanche events being later (~1720 AD) and rockfalls later still (~1740 AD) (Knight & Harrison, 2013). This may be indicative of these different mass movements having different sensitivities to forcing, and thus being triggered by different environmental conditions. This is an important consideration for predicting when and/or where certain mass movements may be found in present mountain environments. Bayesian analysis of debris flows in the French Alps shows that climatic and environmental variables explain 44% and 33% of variance, respectively (Jomelli et al., 2015). A time series of rockfall events in Austria does not show a close relationship to temperature and thus climate, but there is a spring peak in rockfall that likely corresponds to subsurface ice melt at the end of the winter season (Sass & Oberlechner, 2012). However, mass movements can also be generated by individual weather events such as the 2003 European heatwave and 2005 floods (Gruber, Hoelzle & Haeberli, 2004; Keiler, Knight & Harrison, 2010; Bodin et al., 2017). These extreme weather events are predicted to become more common under global warming, especially over mountain regions (Huggel et al., 2010; Ding et al., 2020; Thornton et al., 2021; Adler et al., 2022).

### Mountain ecosystems and services

Mountain (alpine) ecosystems are strongly climatically controlled by direct forcing of mountain temperature and precipitation regimes, and indirectly through climatic influence on soils. As such, mountain ecosystems and ecosystem services are sensitive to climate and environmental disturbance and change, including by human activity (Löffler et al., 2011; Elkin et al., 2013; Mina et al., 2017; Wei et al., 2022). The different physical properties of mountains, including their elevation and remoteness, also provide different ecological niches and can favour endemics. In detail, many mid-latitude mountains that were affected by Pleistocene glaciations have present-day ecosystems that can be considered as ice age relicts or refugia, in which cold-climate ecosystems occupy small environmental niches at the tops of mountains that are particularly climatically sensitive (*e.g*., Muellner-Riehl, 2019). Progressive warming, whether from the lateglacial into the Holocene or during the Anthropocene, results in distinctive trajectories of climate and environmental change on mountains that have implications for ecosystems (Löffler et al., 2011). These include an upslope migration of isotherms, increased number of degree days available for plant growth, longer summer growing season, warmer ground surface temperatures, enhanced biogeochemical cycling, decreased number and intensity of frost days, changes in snowline/treeline position, reduced snow cover thickness and duration, and changed river discharge patterns and water quality (affecting aquatic ecosystems) (Gonzalez et al., 2010; Cauvy-Fraunié & Dangles, 2019; Losapio et al., 2021). These climatic changes then have implications for associated environmental regimes such as soil development and slope stability (Perrigo, Hoorn & Antonelli, 2020). Several studies also show there is a close correspondence between glacier retreat (Cauvy-Fraunié & Dangles, 2019), and permafrost warming as triggers for the altitudinal spread of plant species and thus mountain ecosystem development (Wei et al., 2022).

Detailed analysis shows that different mountain species and biomes exhibit different responses to climate change (Thapa et al., 2016; Albrich, Rammer & Seidl, 2020; Losapio et al., 2021). This includes range shifts and changes in phenology. Most work has been done on forests, because of their implications for carbon (C) storage and timber harvesting in mountains, their role as habitats for other plant and animal species, and their role in land surface stabilisation. Studies on forest biome responses to climate forcing have mainly focused on temperature rather than precipitation (*e.g*., Fischlin & Gyalistras, 1997; Jochner et al., 2017). It may be that the functional water balance is more important in certain altitudinal ranges but that this is more strongly moderated by site-scale topography rather than precipitation alone (Albrich, Rammer & Seidl, 2020). Climate model projections show that, although there is an upward increase in treeline position and thus a general upward zonal migration of alpine forests (Lamsal et al., 2017), this should not be considered as a simple deterministic response to climate warming. This is because it does not account for other factors determining biome responses, such as the role of species’ competition, differential species’ vagility, invasive species, and steeper slopes, thinner soils and increased windiness with elevation, and direct human impacts on land cover types. Differential mobility and adaptive capacity of individual species undergoing climate forcing can result in changes in the overall composition of mountain plant communities and, more widely, of food webs (Malanson et al., 2019). This then poses problems for the ability of entire biomes to respond to climate change with, for example, individuals at the lowest altitudinal range limits being most vulnerable to climate change but exhibiting different inter-species dynamics than those elsewhere within the geographical range (Hampe & Petit, 2005; Iglesias et al., 2018). Likewise, ecosystem services in mountain regions are not well understood compared to other environments (Palomo, 2017; Mengist, Soromessa & Legese, 2020). These ecosystem services may include different biological functions such as gene flow (Fady et al., 2008) and C storage (Millar et al., 2017); economic functions; and regulatory and cultural services (Mina et al., 2017; Seidl et al., 2019). There is less understanding of human interactions with mountain ecosystems when compared with other mountain environmental resources such as water.

Climate models have been used in order to predict future mountain climates and, from this, to use ecological models to examine variations in biome spatial area, ecosystem composition, C storage, disease/pathogen spread, and the viability of certain endangered or invasive species (Fischlin & Gyalistras, 1997; Elkin et al., 2013). Key questions going forward focus on the role of detailed mountain topography and therefore micro-environmental niches for species migration routes (Perrigo, Hoorn & Antonelli, 2020), and the potential for gene flow and survivability of endemics in specific locations (Blanco-Pastor et al., 2019). This highlights the site-specific and species-specific nature of mountain ecosystems and their potential responses to anthropogenic climate change (Gonzalez et al., 2010; Blanco-Pastor et al., 2019). A further question, however, is the role of direct human activity in mountain land use change, in particular related to agriculture and forestry, that can impact on mountain biodiversity and the conservation of endangered alpine species (Gehrig-Fasel, Guisan & Zimmermann, 2007; Seidl et al., 2019).

### Mountain communities and infrastructure

Mountain environments and resources represents a ‘global common good’ made use of by mountain inhabitants and visitors alike (Debarbieux & Price, 2008, 2012; Chakraborty, 2020). As such, people and mountain environments are closely interlinked, through water and food resource use, ecosystems and ecosystem services, and human livelihoods (Martín-López et al., 2019). Mountain agricultural economies have historically been founded on pastoralism and viewed as insular and isolated systems (Tahmasebi, Ehlers & Schetter, 2013), but these are now seen as extending into complex spatial networks comprising other mountain goods and services, including cultural patterns, and existing over long time periods (Spies, 2018; Said et al., 2019). Although also a product of more recent globalization, changes in human activities in mountains (agriculture, tourism, industry) are influenced by climate change through changing ecosystems and snow distributions. This is framed through the lens of socioecological vulnerability and resilience (Pandey & Bardsley, 2015; Nettier et al., 2017; Kumar, Fürst & Joshi, 2021) which describe the co-relationships between mountain environments/resources and different human activities. Fraser, Mabee & Slaymaker (2003) term this *environmental sensitivity* and *social resilience*, respectively. Several recent studies have discussed these elements in different sectors of the Himalayas (Kaul & Thornton, 2014; Chettri, Shrestha & Sharman, 2020; Kumar, Fürst & Joshi, 2021) and highlight the importance of integrated hazard risk management and adaptive planning at the community level and with the involvement of indigenous knowledge systems. However, such an approach to minimising climate change risks in mountains has not yet been widely developed for different mountain ranges (*e.g*., McDowell et al., 2019; Payne et al., 2020). An exception is the study by Hossain et al. (2020) that describes the feedbacks that exist within and between the socioeconomic and biophysical systems of rural communities in the Swiss Alps.

The most significant issue affecting people and communities in and downstream of mountains is changes in glacier- and snow-fed river discharge (Viviroli & Weingartner, 2004; Milner et al., 2017; Li et al., 2020). Such mountain ‘water towers’ contribute significantly to regional water supply to, for example, around 60 million people within the Indus and Brahmaputra catchments (Immerzeel, van Beek & Bierkens, 2010), and in turn on regional food security (Carey et al., 2017; Spies, 2018). Based on a global topographic dataset, Viviroli et al. (2007) showed that 43% of mountain areas provide essential or supportive water resources for mainly urban populations, in particular during the dry season and in semiarid areas such as in central Asia. Schaner et al. (2012) estimated that 370 million people globally reside in catchments where glacier melt represents one tenth of seasonal river discharge, and 140 million people in catchments where glacier melt contributes one quarter of total river discharge. Enhanced glacier melt under global warming is progressively both increasing and causing more variability of river discharge (Juen, Kaser & Georges, 2007). Several studies now identify the multiple ways in which mountain water sources impact on people (economy, culture, infrastructure, hydropower, food/water security) and the environment (geohazards, irrigation, ecosystems) (Mukherji et al., 2015; Carey et al., 2017; Hill et al., 2017). These are key areas of research interest because of the intersectionality between people and the environment in mountains, and with reference to sustainable development, and the nexus between food, water and energy security (Rasul, 2014). Further, based on climate model results, it is likely that continued glacier melt over the next decades will result in progressively lower and more variable discharges as glacier volume decreases (Messerli, Viviroli & Weingartner, 2004; Juen, Kaser & Georges, 2007). This has implications for sediment yield and geohazards, as well as water supply (Knight & Harrison, 2013; Mukherji et al., 2015; Milner et al., 2017) and water management strategies (López-Moreno, Beniston & García-Ruiz, 2008; Bombelli et al., 2019). Contemporary snow and glacier retreat in mountains is already impacting on the development and sustainability of mountain tourism and conservation of the natural environment (Purdie, 2013; Pröbstl-Haider, Dabrowska & Haider, 2016; Su et al., 2022) and its built heritage (Duvillard et al., 2019).

## Discussion

Mountain environments today are in a state of rapid transition as a consequence of climate change in the Anthropocene (Gerrard, 1991; Marston, 2008; Milner et al., 2017; Rasul & Molden, 2019). This study sends a powerful Warning to Humanity regarding the ways in which anthropogenic climate change negatively impacts on mountains and the people who reside in them, through the workings of social-ecological and physical systems. Many case studies from the world’s mountains highlight the critical risks that climate change impacts pose for regional food, water and energy security, the maintenance of biodiversity and infrastructure, and the preservation of cultural heritage (*e.g*., Rasul, 2014; Pandey & Bardsley, 2015; Chakraborty, 2020; Hossain et al., 2020). Addressing these issues through adaptation and mitigation, and monitoring and modelling of mountain system dynamics, is critical for future sustainability of these joint human–physical systems, and for water security for millions of people (Hill et al., 2017; Milner et al., 2017; Li et al., 2020).

**Figure 2:**
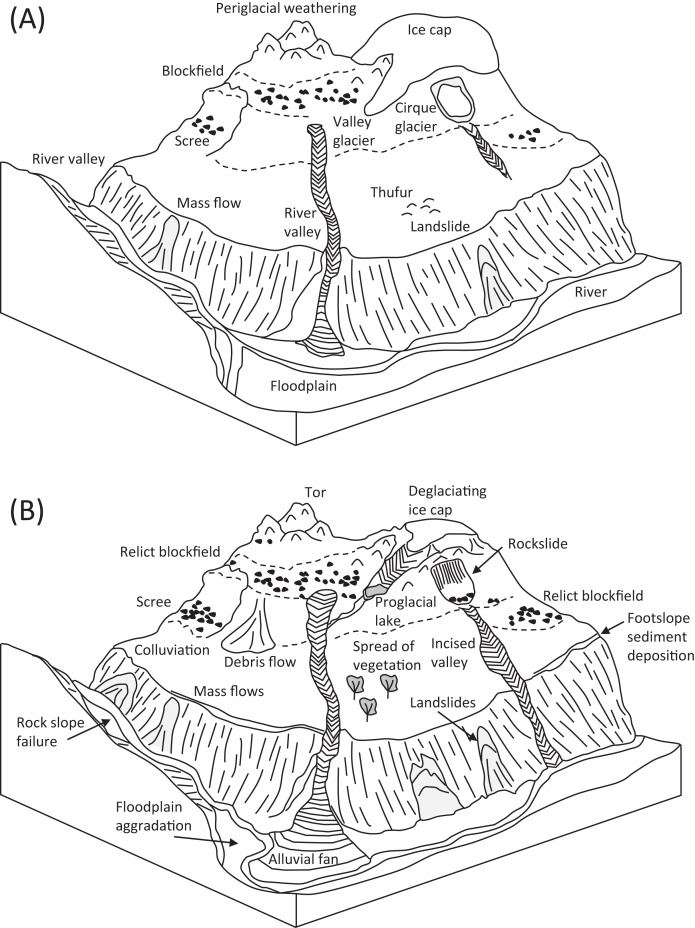
Schematic block diagrams illustrating the geomorphic patterns and processes taking place in mountains under (A) pre-Anthropocene, and (B) Anthropocene climates associated with a decline in the mountain cryosphere (sketches not to scale).

Figure 2 qualitatively illustrates the major biophysical properties of mountain landscapes and their likely future changes under ongoing climate change. Key elements of these landscapes include glacial and periglacial landforms and processes in highest altitudes, with mass movements on lower slopes, and aggradation within river valleys (Knight & Harrison, 2009). Warming climates give rise to spatial variations in mountain process domains, with glacial and periglacial areas shrinking, and slope instability reflecting paraglaciation increasing in prominence (Knight & Harrison, 2013). Several modelling studies suggest total deglacierization of some mountain sectors, along with spread of ecosystems, over coming decades (Zemp et al., 2006; Rabatel et al., 2018). This represents a fundamental first-order change in the operation of mountain systems, on a global scale (Milner et al., 2017). The full implications of this have yet to be realized through field or modelling studies, but include regional heat balance and climate (including impacts on monsoon circulation), biogeochemical cycling and hydrological balance. Full impacts on people—including mountain dwellers and those within mountain-sourced river catchments—have also yet to be realized, and this is important for developing adaptation strategies for future changes in both mountain geohazards and mountain socioeconomic and cultural systems (Chakraborty, 2021).

Several conceptual frameworks have been developed to better understand the workings of integrated mountain systems. A *biophysical systems* approach can be used to conceptualise relationships between the different biological, geomorphological and climatic elements that exist within mountain systems (Hossain et al., 2020). Most previous work on biophysical systems in mountains has focused on ecosystem processes and drivers such as fire regime (*e.g*., Argañaraz et al., 2015; Zapata-Ríos et al., 2021) and their implications for ecosystem and species’ dynamics (*e.g*., Zhang et al., 2018; Davis et al., 2021). Fewer studies have examined the specific genetic linkages that exist between ecosystems and the physical environment itself (soils and substrate type, permafrost distribution) (Bugmann et al., 2007; Xu et al., 2008; Ran et al., 2021). These are important, however, because ecosystems are dependent upon substrate and climatic properties, and these in turn then link to the provision of different ecosystem services, in particular through agriculture (Bagstad et al., 2016; Zhang et al., 2021). The conceptual analysis of human activity in mountain landscapes has also commonly been undertaken through the lens of *socio-ecological systems* (*e.g*., Hossain et al., 2020; Berrio-Giraldo, Villegas-Palacio & Arango-Aramburo, 2021; Fernández-Giménez et al., 2021; Grumbine & Xu, 2021; Gopirajan, Kumar & Joshi, 2022) but this approach deals only with human interactions with mountain environments, not with changes in those environments because of climate and associated human adaptive responses. Thus, both biophysical and socio-ecological systems’ approaches have some limitations when applied to mountain environments, and lack integration. For this reason, here the portmanteau term *socio-biophysical systems* is introduced to describe the nature of human–environment relations in mountains (Fig. 2). Hossain et al. (2020) considered some of the feedbacks that exist between human and biophysical systems, based on examples from rural communities in the Swiss Alps. They developed a ‘mountain community coupled human landscape system’ model (*e.g*., Alberti et al., 2011) to explain these relationships but with an emphasis on geohazard risk and mitigation rather than understanding the workings of mountain systems.

**Figure 3:**
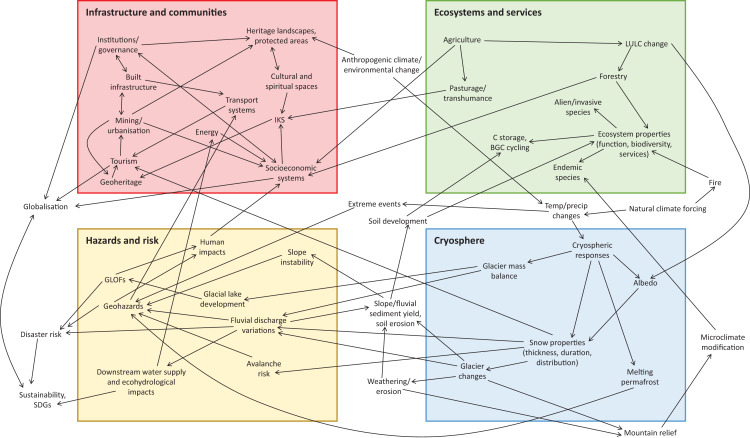
Interlinkages of different physical and human mountain elements, grouped under the four themes identified in this study, within a socio-biophysical model. Abbreviations are: LULC, land use/land cover; IKS, indigenous knowledge systems; GLOFs, glacial lake outburst floods; SDGs, Sustainable Development Goals.

Figure 3 proposes a socio-biophysical systems model to describe and account for the co-relationships between different constituents of mountain systems, including the key transformative role of human activity and anthropogenic climate change in the Anthropocene. The model is organized according to the four thematic areas identified in the literature review of this study, and it highlights that there are multiple interconnections between different mountain elements that cross between these thematic areas. The elements described in this model build from and extend the limited socio-ecological connections identified in previous studies (*e.g*., Alberti et al., 2011; Hossain et al., 2020; Kumar, Fürst & Joshi, 2021). Figure 3 identifies that there are a number of items that cross different thematic areas, thereby demonstrating interconnections between socio-ecological and biophysical systems. These include anthropogenic climate/environmental change, physical landscape processes, land use/land cover change, geohazards, and tourism. Some of these elements have been included in some previous evaluations of socio-ecological and biophysical mountain systems (*e.g*., Bugmann et al., 2007; MacMynowski, 2007; Hill et al., 2017; Hossain et al., 2020; Payne et al., 2020; Kumar, Fürst & Joshi, 2021; Gopirajan, Kumar & Joshi, 2022), but some have not. The interconnections existing within this model also speak to the potential resilience and vulnerability exhibited by both human and environmental systems in mountains, whereby the negative impacts of ongoing changes within mountains can be mitigated. Understanding these interrelationships, including community adaptations to environmental change in mountains, is an important research priority (Gentle & Maraseni, 2012; Grumbine & Xu, 2021; Kumar, Fürst & Joshi, 2021).

## Conclusions

Mountain systems are sensitive to global warming in the Anthropocene, and thus it is timely that a Warning to Humanity is issued, highlighting the serious negative impacts of global warming and associated societal responses for mountain environments and communities, both within mountain massifs and in their extensive surrounding hinterlands. A systems approach, considering and integrating together the different properties of mountain environments, is a useful framework for examining mountain environment dynamics (Fig. 3). The impacts of climate warming, ice retreat and associated changes in the properties and dynamics of mountain systems have been widely examined from local case-studies (*e.g*., Gude & Barsch, 2005; Singh, 2009; Gariano & Guzzetti, 2016), but more work is needed to understand the spatial contingency of geohazards and therefore geohazard risk that arise as a consequence of climate change. This is an important future research priority (Tullos et al., 2016). Likewise, the impacts of environmental change on (often vulnerable) mountain communities, and their societal and socioeconomic responses, have also been examined from some locations (*e.g*., Carey et al., 2017; Rasul & Molden, 2019) but many mountains especially in the developing world have not yet been considered (Yohannes, Teshome & Belay, 2020). These are also important research priorities because they focus on building community adaptation and resilience (Gentle & Maraseni, 2012; Xenarios et al., 2019; Hossain et al., 2020; Grumbine & Xu, 2021).

Achieving sustainable development in mountains requires a deeper understanding of the interactions between human activity and the physical environment in mountains (Klein et al., 2019; Payne et al., 2020). Conserving and managing mountain sociocultural and biosystems are specifically mentioned in the 2030 Agenda for Sustainable Development and in Chapter 13 of Agenda 21. Many local case studies, in particular in the Himalayas, have examined interrelationships between physical environmental change and community adaptations to challenges posed by water availability, hazards, agriculture, and ecosystem services (Gentle & Maraseni, 2012; Sujakhu et al., 2019). However, equivalent data are often lacking for many other mountain blocks worldwide. The proposed socio-biophysical systems model (Fig. 3) provides a global framework for a better understanding of the dynamics of mountains in the 21^st^ century, affected by climate change and increased human impacts. This highlights why a Warning to Humanity on the sensitivity of mountain systems to climate change and environmental disturbance in the Anthropocene is important and timely.

## Additional Information and Declarations

### Data Availability

The following information was supplied regarding data availability: There is no raw data in this literature review.
